# Combining Probability and Nonprobability Samples by Using Multivariate Mass Imputation Approaches with Application to Biomedical Research

**DOI:** 10.3390/stats6020039

**Published:** 2023-05-08

**Authors:** Sixia Chen, Alexandra May Woodruff, Janis Campbell, Sara Vesely, Zheng Xu, Cuyler Snider

**Affiliations:** 1. Department of Biostatistics and Epidemiology, University of Oklahoma Health Sciences Center, 801 NE 13th St, Oklahoma City, OK 73104, USA; 2. Department of Mathematics and Statistics, Wright State University, Dayton, Ohio 45324, USA; 3. Southern Plains Tribal Health Board, 9705 Broadway Ext, Oklahoma City, OK 73114, USA

**Keywords:** nonprobability sample, multivariate imputation, public health data, selection bias

## Abstract

Nonprobability samples have been used frequently in practice including public health study, economics, education, and political polls. Naïve estimates based on nonprobability samples without any further adjustments may suffer from serious selection bias. Mass imputation has been shown to be effective in practice to improve the representativeness of nonprobability samples. It builds an imputation model based on nonprobability samples and generates imputed values for all units in the probability samples. In this paper, we compare two mass imputation approaches including latent joint multivariate normal model mass imputation (e.g., Generalized Efficient Regression-Based Imputation with Latent Processes (GERBIL)) and fully conditional specification (FCS) procedures for integrating multiple outcome variables simultaneously. The Monte Carlo simulation study shows the benefits of GERBIL and FCS with predictive mean matching in terms of balancing the Monte Carlo bias and variance. We further evaluate our proposed method by combining the information from Tribal Behavioral Risk Factor Surveillance System and Behavioral Risk Factor Surveillance System data files.

## 1. Introduction

A probability sample is formally defined as the sample obtained by the probability sampling design such that each unit in the target population will have a non-zero probability of being selected. On the other hand, a nonprobability sample is the opposite, such that not every unit in the target population will have a non-zero probability of being selected [1]. A probability sample has attractive theoretical properties including low selection bias and representativeness [1]. A nonprobability sample may produce biased results without further adjustment [2]. Even though probability sampling has been regarded as the gold standard for obtaining representative information for the target population of interest [3,4], nonprobability sampling (e.g., convenient sample) has been used frequently in practice including public health study, economics, education, and political polls due to its cost and time efficiency and the lack of a sampling frame [5]. For instance, the sampling frame for studying HIV patients is not available when drawing a probability sample. Furthermore, the quality of probability samples has decreased rapidly in recent years due to a decline in the response rate of recent surveys [6]. In addition, the availability of low-cost big data obtained from social media, web, and panel surveys makes the nonprobability sample more attractive nowadays [7].

Nonprobability samples have been used frequently in practice. To name a few, Pew Research Centre (http://www.pewresearch.org) provides a 2015 dataset consisting of nine nonprobability samples with a total of 9301 individuals and a wide range of measurements over 56 variables related to economics, social economics, and health behaviors. The 2019 Tribal Behavioral Risk Factor Surveillance System (TBRFSS) survey, conducted by Oklahoma Tribal Epidemiology Center, used a mix of convenience sampling by attending tribal events in person, over email, and through website availability [8]. The TBRFSS collects health-related information for the Native American populations living in Kansas, Oklahoma, and Texas. As another example, [9] estimated the national Criminal Justice Attitudes from five online nonprobability samples drawn either from Amazon Mechanical Turk or an opt-in panel. In summary, nonprobability samples include all types of samples where a random selection process is lacking.

Even though nonprobability samples have been used frequently in practice, naïve estimates based on them, without proper adjustments, may lead to biased results [2] due to selection bias. Currently, most population approaches for handling nonprobability samples include calibration, propensity score weighting, mass imputation, and hybrid methods. All those methods were designed to combine the information from the nonprobability sample and probability sample, and they all assume that there are some overlapping covariate variables between the two samples, which is very common in practice. Calibration methods [10,11] produce calibrated weights in the nonprobability sample such that the weighted frequency or total by using the nonprobaiblity sample with calibrated weights benchmark with the weighted frequency or total by using the probability sample with final weigh. The underlying assumption for the calibration method is that there is a linear association between the outcome variables of interest and overlapping covariate variables. Propensity score weighting methods [12,13] produce models for the selection mechanism in the nonprobability sample and estimate the selection probability by solving the estimating equations which link information from two samples. The validity of the propensity score methods depends on the underlying model assumptions for the selection mechanism. Mass imputation methods [14–16] first build imputation models by using nonprobability sample and then generate imputed values of outcome variables based on the probability sample and fitted imputation models. The validity of mass imputation methods depends on the underlying imputation models. To protect the model misspecification, hybrid methods [17,18] were developed by using both imputation models and propensity score models so that the estimates are consistent if one of those models is corrected specified.

Although many data integration methods have been developed for combining probability and nonprobability samples, the discussion of multivariate mass imputation methods is sparse, except for a recent paper [8] by us which applies a fully conditional specification (FCS) procedure [19,20] to combine the Tribal Behavioral Risk Factor Surveillance System (TBRFSS) and Behavioral Risk Factor Surveillance System (BRFSS). We showed that the FCS procedure outperformed naïve estimates without any adjustment in terms of bias for nine health outcome variables. However, the comparison between the FCS procedure and a newly developed procedure called latent joint multivariate normal model mass imputation (e.g., Generalized Efficient Regression-Based Imputation with Latent Processes (GERBIL)) [21] in both a Monte Carlo simulation study and real application is lacking in the existing literature. In a recent study [21] on the missing data problem, GERBIL has been shown to have more attractive properties than the FCS procedure. Multivariate mass imputation is important in practice since researchers need to conduct a statistical analysis for multiple outcome variables in the study simultaneously. In this paper, we fill this important research gap by using both a Monte Carlo simulation study and real application with TBRFSS and BRFSS data files. In addition, computation codes were developed for other researchers to use.

The rest of the paper is organized as follows: Section 2 presents data files, variables, and our proposed methods. The Monte Carlo simulation results as well as those of the real application are included in Section 3. Section 4 contains the summary and conclusion.

## 2. Materials and Methods

### 2.1. Multivariate Mass Imputation Approaches

We briefly introduce the following two multivariate mass imputation approaches in this section: fully conditional specification (FCS) procedure and Generalized Efficient Regression-Based Imputation with Latent Processes (GERBIL). Suppose we have a finite population ${𝒡}_{N}=\{\left(X_{i},Y_{i}\right),\;i=1,2,\ldots N\}$ with $X_{i}=(X_{i,1},X_{i,2},\ldots X_{i,p})$ as the covariate vector with dimension *p*, $Y_{i}=(Y_{i,1},Y_{i,2},\ldots Y_{i,q})$ as the study variable vector of interest with dimension q, and N is the population size. Assume we have a probability sample $S_{A}$ with sampling weight $w_{i}$ for unit $i\in S_{A}$ and we only observe $X_{i}$ in $S_{A}$. Denote $S_{B}$ as a nonprobability sample and assume both $X_{i}$ and $Y_{i}$ are observed in $S_{B}$. This setting is consistent with practical scenario. We assume the same mass imputation model $f(Y_{i}|X_{i})$ holds for both probability sample $S_{A}$ and nonprobability sample $S_{B}$. This is similar to missing at random assumption. For simplicity, suppose the parameter of interest is the population mean of Y, which can be written as $\theta_{N}=N^{-1}\sum_{i=1}^{N}Y_{i}$. The idea for FCS is that one can first generate initial imputed values of $Y_{i}$ for all observations in probability sample $S_{A}$, then one can conduct sequential imputation by using the combined data file of $S_{B}$ and $S_{A}^{*(0)}$ and conditional model $f(Y_{i,j}|X_{i},Y_{i}^{*(-j)})$ for each item $Y_{i,j}$ for $j=k_{1},k_{2},\ldots k_{q}$ where $k_{1},k_{2},\ldots k_{q}$ is a pre-specified order of 1,2,…q, where $Y_{i}^{*(-j)}$ is an imputed study variable vector from previous iteration after excluding the item $Y_{i,j}$. Then, one should repeat the previous sequential imputation process a large number of times until there is convergence of the imputation. Instead of using conditional distribution for sequential imputation, the imputation process for GERBIL relies on joint multivariate distribution $f(Y_{i}|X_{i})$. GERBIL models this joint distribution by using Gaussian Latent Processes first, and then it generates imputed values to all observations in the probability sample $S_{B}$ simultaneously. For more detailed technical information, please refer to [21]. After the mass imputation by either of the previous two methods, the mass-imputed estimator can be written as $\hat{\theta }={\hat{N}}^{-1}\sum_{i\in S_{A}}w_{i}Y_{i}$ , where $\hat{N}=\sum_{i\in S_{A}}w_{i}$. Practically, the FCS has advantage in terms of modeling flexibility since it is relatively easier to model the conditional distribution for unit study item instead of the joint distribution of study variable vector. Theoretically, GERBIL method has advantage since the theoretical proof of recovering the joint distribution from conditional distribution is lacking.

### 2.2. Monte Carlo Simulation Study

We generated M=1000 Monte Carlo samples. In each sample, we first generated one finite population with size N=10,000 with seven variables $X_{1},\;X_{2},\ldots X_{7}$ generated from the following super-population models. $X_{1}$ was generated from a multinomial distribution with 3 categories (1, 2, and 3) of probabilities $\left\{0.2,0.3,0.5\right\}$. Denote $Z=(Z_{2},Z_{3},Z_{4},Z_{5},Z_{6})$ as the unobserved latent variables which were generated from a multivariate normal distribution with mean vector $\mu =\left(X_{1},X_{1}+3,\frac{X_{1}}{3},X_{1}+2,2X_{1}-3\right)$ and covariance matrix with diagonal elements equal to 1 and off-diagonal elements equal to 0.5. Let $X_{2}=Z_{2},\;X_{3}=Z_{3},\;X_{7}=Z_{6}\;X_{4}$ be a dummy variable (1 or 0) for event $Z_{4}\geq 0.5$, and $X_{5}$ be a three level categorical variable such that $X_{5}=1$ if $Z_{5}\leq 3,\;X_{5}=2$ if $3<Z_{5}\leq 5$, and $X_{5}=3$ if $Z_{5}\geq 5.\;X_{6}$ was generated from logistic regression model with $logit(p)=X_{1}-X_{2}^{2}-X_{3}+X_{4}+2X_{5}$, where p is the probability for $X_{6}=1$ and $logit\left(p\right)=log(p/(1-p))$ is the logit function. For each finite population, we selected a probability sample A with sample size $n_{A}=500$ by using simple random sampling without replacement and a nonprobability sample B with expected sample size $E(n_{B})=500$ by using Poisson sampling with selection probability depends on $X_{1},\;X_{2}$, and $X_{3}$. For evaluation purposes, we assumed that $X_{1},\;X_{2}$, and $X_{3}$ were observed in both probability sample A and nonprobability sample B, and $X_{4},\;X_{5},\;X_{6}$, and $X_{7}$ were only observed in nonprobability sample B.

We considered the following multivariate mass imputation approaches: (1). Sequential multiple imputation methods with 10 imputations. In [22], it was suggested that it is sufficient to use 5 to 10 imputed values for multiple imputation in practice. We used R package ‘mice’ with method=‘pmm’, ‘cart’, and ‘rf’. (2). Generalized Efficient Regression-Based Imputation with Latent Processes. We used R package ‘gerbil’ with 10 imputations. For evaluating the selection bias of nonprobability sample, we first compared the distributions of all variables among population, probability sample, and nonprobability sample. Then, Monte Carlo biases were calculated for comparing the two methods for estimating the population means of $X_{4},\;X_{5},\;X_{6}$, and $X_{7}$.

### 2.3. Real Data Application

In this section, we compared the two multivariate mass imputation methods described in the Monte Carlo simulation study section by using real data files. Specifically, we used 2018 and 2019 Behavioral Risk Factor Surveillance System (BRFSS) surveys as the probability sample. BRFSS is a national level probability-based dual-frame (e.g., Cell and Landline) telephone sample. It collects health-related information for US adults. Weighting procedures including nonresponse adjustment and calibration were performed by Centers for Disease Control and Prevention (CDC) for reducing the selection bias of BRFSS. To make the combined sample representative, we combined 2018 and 2019 BRFSS samples by using composite weighting procedure [23–25]. After combining, we had about 970 Native American adults in Oklahoma state. We used 2019 Tribal Behavioral Risk Factor Surveillance System (TBRFSS) survey as the nonprobability sample. TBRFSS survey is a convenient sample collected by using a combination of event sampling, email sampling, and social media sampling. It collects health-related information for Native American adults in Oklahoma, Kansas, and Texas. The sample size for Native American adults in Oklahoma state is 747.

There are many overlapping variables in BRFSS and TBRFSS, which provides an idea data source for evaluation of the two multivariate mass imputation approaches for data integration. For evaluation purposes, we considered the following eight covariate variables since they were observed in both data files: Age Group, Gender, Marital Status, Education Level, Employment Status, Income Level, BMI Status, and General Health Status. In addition, we considered the following six health-related study variables: Smoking status, Cardiovascular Disease status (CVD), Asthma status, Stroke status, Diabetes status, and Health Coverage status. Even though the six study variables were observed in both data files as well, we assumed that they were only observed in TBRFSS, then use the information in BRFSS as the benchmark to calculate the bias. Our parameters of interest are the population prevalence of the above six health-related study variables.

## 3. Results

### 3.1. Monte Carlo Simulation Study

Table 1 presents the comparison of weighted averages based on probability sample, unweighted averages based on nonprobability sample, and the population averages based on finite population for the seven variables. As expected, the weighted averages based on probability sample are very close to the population averages since they are unbiased estimates theoretically. The unweighted averages based on the nonprobability sample are quite different from the population averages due to selection bias. According to Table 2, multivariate mass imputation methods based on mice (pmm) and gerbil outperform other methods in terms of biases for all variables. Almost all multivariate mass imputation methods outperform the unweighted average based on the nonprobability sample in Table 1, which shows the benefits and validity of the multivariate mass imputation procedures for data integration.

### 3.2. Real Data Application

Table 3 presents the comparison between distributions for eight covariate variables defined previously between BRFSS and TBRFSS. According to Table 3, there is a large discrepancy between the weighted frequency (percentage), using BRFSS, and the unweighted frequency (percentage), using TBRFSS. For example, the weighted percentage for the 18–24 age group based on BRFSS is 17.07%, and the unweighted percentage for the same age group based on TBRFSS is only 5.83%. The weighted percentage for Male based on BRFSS is 48.80%, and the unweighted percentage for Male based on TBRFSS is only 22.05%. We used the Rao–Scott Chi-square test to test the significance of such a discrepancy for each variable, and it transpires that all results are significant with p values less than 0.001. Such a large discrepancy indicates the large selection bias of using TBRFSS without further adjustment. Table 4 presents the biases for estimating nine study variables by using different multivariate mass imputation methods. The best method for estimating each study variable is highlighted in bold. According to Table 4, all multivariate mass imputation methods outperform the unweighted Naïve methods by using TBRFSS in terms of biases. Mice and gerbil methods have comparable results in general. The mice (rf) method had the best performance for estimating three study variables, but it has the largest biases for estimating the other three study variables. Mice (pmm), mice (cart), and gerbil methods had more stable results. In practice, researchers only considered naïve methods by using unweighted TBRFSS data file only. In this application, we were the first to show the advantages of multivariate mass imputation methods. In addition, we were the first to compare different multivariate mass imputation methods and provide empirical evidence for other researchers.

## 4. Conclusion

Nonprobability samples have been used frequently in biomedical research due to their convenience, lack of sampling frame, low cost, and efficiency in data collection in the nonprobability sampling design. Specifically, the sampling frames for most diseases are not available for researchers to draw probability samples. However, most biomedical studies still used naïve estimates from nonprobability samples without any further adjustment, which may lead to biased results for the target population of interest. Studies conducted at one time and location are not generalizable to studies which will be conducted at another time or location. Data integration is an important research question for public health study due to the frequent use of nonprobability samples and the availability of high-quality large-scale probability samples. Statistical analysis solely based on nonprobability samples may lead to biased results due to the selection bias of nonprobability samples. The mass imputation procedure has been shown to be one of the most effective data integration methods for combining information from probability and nonprobability samples. However, multivariate mass imputation approaches have not been well studied in the existing literature. In this paper, we filled an important research gap by comparing two multivariate mass imputation approaches (e.g., mice and gerbil methods) by using both a simulation study and real data application. Both the simulation study and real data application showed that the two mass imputation methods reduced selection biases compared with the naïve method by only using the nonprobability sample. In the simulation study, multivariate mass imputation methods based on mice (pmm) and gerbil outperform mice (cart) and mice (rf) in terms of biases for all variables. In the real data application, mice (rf) outperformed other methods for estimating three study variables, but it has the largest biases for estimating the remaining study variables. Mice (pmm), mice (cart), and gerbil methods had more stable performance for estimating all study variables. In terms of limitations, we only considered some commonly used methods for mice including regression tree and random forest. There might be other applicable machine learning methods, including support vector machine, deep neural networks, and many others. However, due to the limitation of existing computational tools, we only considered a few in the paper. In terms of future research directions, it might be interesting to conduct a more empirical comparison by using a greater number of real data applications. Statistical inference, including variance estimation, hypothesis testing, and confidence interval after multivariate mass imputation, is also an important future research topic. Lastly, it might be interesting to investigate other machine learning-based mass imputation approaches described above.

## Figures and Tables

**Table 1.** Comparison of population averages, probability sample weighted averages, and nonprobability sample unweighted averages.

Variable	Population	Probability Sample	Nonprobability Sample
X1 (Value=1)	0.200	0.199	0.041
X1 (Value=2)	0.300	0.299	0.077
X2	2.300	2.301	2.836
X3	5.300	5.298	5.988
X4	0.602	0.602	0.688
X5 (Value=1)	0.159	0.159	0.049
X5 (Value=2)	0.538	0.538	0.478
X6	0.303	0.304	0.336
X7	1.600	1.602	2.727

**Table 2.** Comparison of multivariate mass imputation methods based on Monte Carlo simulation.

Variable	Method	Estimate	Bias
X4	mice (pmm)	0.598	−0.0036
	mice (cart)	0.573	−0.0288
	mice (rf)	0.706	0.1041
	gerbil	0.603	0.0012
X5 (Value=1)	mice (pmm)	0.159	0.0002
	mice (cart)	0.140	−0.0188
	mice (rf)	0.048	−0.1112
	gerbil	0.160	0.0012
X5 (Value=2)	mice (pmm)	0.537	−0.0007
	mice (cart)	0.543	0.0052
	mice (rf)	0.569	0.0314
	gerbil	0.534	−0.0039
X6	mice (pmm)	0.312	0.0091
	mice (cart)	0.282	−0.0213
	mice (rf)	0.269	−0.0339
	gerbil	0.308	0.0049
X7	mice (pmm)	1.603	0.0025
	mice (cart)	1.603	0.0025
	mice (rf)	1.603	0.0025
	gerbil	1.603	0.0025

**Table 3.** Comparison of distributions of covariate variables in BRFSS and TBRFSS.

Variable	Value	BRFSS weighted Frequency (Percent)	TBRFSS unweighted Frequency (Percent)
age*	18–24	46,597 (17.07)	37 (5.83)
	25–29	30,027 (11.00)	48 (7.56)
	30–34	32,567 (11.93)	46 (7.24)
	35–39	29,459 (10.79)	49 (7.72)
	40–44	19,838 (7.27)	55 (8.66)
	45–49	17,961 (6.58)	51 (8.03)
	50–54	21,637 (7.93)	63 (9.92)
	55–59	21,303 (7.81)	94 (14.80)
	60−64	16,142 (5.91)	76 (11.97)
	65–79	12,267 (4.49)	59 (9.29)
	70+	25,129 (9.21)	57 (8.98)
gender*	Male	133,198 (48.80)	140 (22.05)
	Female	139,728 (51.20)	495 (77.95)
marital*	Married	120,946 (44.31)	242 (38.11)
	Divorced/Separated	50,397 (18.47)	142 (22.36)
	Widowed	16,701 (6.12)	60 (9.45)
	Never Married	72,022 (26.39)	114 (17.95)
	Member of unmarried Couple	12,861 (4.71)	77 (12.13)
education*	Less than High School	38,116 (13.97)	63 (9.92)
	High School Graduate	103,878 (38.06)	191 (30.08)
	Some college/technical school	89,158 (32.67)	231 (36.38)
	College Graduate	41,774 (15.31)	150 (23.62)
employ*	Employed/Self-employed	157,742 (57.80)	400 (62.99)
	Unemployed/Homemaker/Student	49,507 (18.14)	72 (11.34)
	Retired	31,124 (11.40)	104 (16.38)
	Unable to Work	34,553 (12.66)	59 (9.29)
income*	Less than USD 10,000	24,554 (9.00)	117 (18.43)
	Less than USD 15,000	11,586 (4.25)	60 (9.45)
	Less than USD 20,000	32,404 (11.87)	63 (9.92)
	Less than USD 25,000	29,114 (10.67)	76 (11.97)
	Less than USD 35,000	35,740 (13.10)	88 (13.86)
	Less than USD 50,000	42,416 (15.54)	89 (14.02)
	Less than USD 75,000	40,524 (14.85)	79 (12.44)
	USD 75,000 or More	56,587 (20.73)	63 (9.92)
BMI Cat*	Underweight/Healthy weight	64,439 (23.61)	105 (16.54)
	Overweight	98,507 (36.09)	176 (27.72)
	Obese	109,980 (40.30)	354 (55.75)
general health*	Excellent	37,839 (13.86)	56 (8.82)
	Very Good	78,767 (28.86)	144 (22.68)
	Good	85,727 (31.41)	261 (41.10)
	Fair/Poor	70,593 (25.87)	174 (27.40)

significant results with p values less than 0.001 are marked with *

**Table 4.** Comparison of multivariate mass imputation methods based on real data application.

Variable	Naïve	mice (pmm)	mice (cart)	mice (rf)	gerbil
cvd	0.0353	0.0434	0.0290	**−0.0081**	0.0401
asth	−0.0300	−0.0273	−0.0405	−0.0867	**−0.0179**
hlthcov	−0.1391	−0.1548	−0.1012	**−0.0535**	−0.1197
stroke	−0.0082	**−0.0015**	0.0033	−0.0334	−0.0027
diabete	0.1070	0.0508	0.0667	**0.0252**	0.0515
smoke	−0.0732	**0.0154**	0.0188	−0.1343	0.0400

## Data Availability

BRFSS data file was obtained from the following publicly available website: https://www.cdc.gov/brfss/index.html TBRFSS is not publicly available and it is confidential data file belongs to Southern Plains Tribal Health Board.
